# Absolute Configuration Determination of Retroflexanone Using the Advanced Mosher Method and Application of HPLC-NMR

**DOI:** 10.3390/md16060205

**Published:** 2018-06-12

**Authors:** Caleb Singleton, Robert Brkljača, Sylvia Urban

**Affiliations:** School of Science (Applied Chemistry and Environmental Science), RMIT University, GPO Box 2476, Melbourne, VIC 3001, Australia; caleb.taicho@gmail.com (C.S.); robert.brkljaca@rmit.edu.au (R.B.)

**Keywords:** Mosher, retroflexanone, phloroglucinol, HPLC-NMR, secondary alcohol

## Abstract

The absolute configuration of retroflexanone (**1**) and a closely related phlorogluinol (**2**) was established using the advanced Mosher method and by application of HPLC-NMR. HPLC-NMR permitted a small scale Mosher method analysis to be carried out on these unstable phloroglucinols.

## 1. Introduction

Retroflexanone (**1**) was recently reported from the dichloromethane extract of *Cystophora retroflexa* [1] using a combination of HPLC-NMR and HPLC-MS [2] while undertaking a phytochemical study of various southern Australian marine algae. Retroflexanone (**1**) contains a single stereogenic centre at the secondary alcohol, but to date, no efforts to confirm its absolute configuration have been undertaken. The Mosher ester analysis which was superseded by the advanced (or modified) Mosher ester analysis became a standard spectroscopic method for the determination of the absolute configuration of secondary alcohols [3,4,5,6,7]. The method relies upon comparing the changes and differences in chemical shifts observed in the ^1^H NMR spectra of the prepared Mosher esters to those of the original natural product containing the secondary alcohol [3,5]. By observing the chemical shifts of each proton in the original secondary alcohol and each of the two prepared esters, the δ*^SR^* value is able to be calculated for each individual proton. This value is simply the chemical shift of the protons in the natural product that is reacted with (*R*)-MTPA (which results in the *S*-MTPA derivative) subtracted from the chemical shift of the protons in the natural product that is reacted with (*S*)-MTPA (which results in the *R*-MTPA derivative). The changes in chemical shifts, together with consideration of the Cahn-Ingold-Prelog priority rules, allow for the absolute configuration to be elucidated.

Preliminary work carried out on this and related marine algae [1,8] indicated that retroflexanone (**1**) was significantly unstable and would most likely be unable to be purified directly from the crude extract in large quantities to perform the traditional advanced Mosher ester analysis.

In an attempt to address the supply and stability issues of retroflexanone (**1**), it was proposed to undertake the advanced Mosher method of retroflexanone, which was present in an enriched fraction, and then conduct HPLC-NMR to determine the absolute configuration by noting the changes in the ^1^H NMR chemical shifts. The advanced or modified Mosher method has previously been carried out using HPLC-NMR in cases where only small amounts of compound were available [9,10,11]. In these instances, small amounts of compound were reacted with the Mosher reagents, and then subsequently purified, and ^1^H NMR data acquired using HPLC-NMR.

This current work represents the fourth instance of the use of HPLC-NMR being used in conjunction with the advanced Mosher method to deduce the absolute configuration of a natural product [9,10,11]. The absolute configuration of the previously reported phloroglucinol (**2**) was determined by application of the advanced Mosher method on an enriched fraction followed by HPLC-NMR analysis of the mixture. The absolute configuration of retroflexanone (**1**) was determined after isolation of small quantities, application of the advanced Mosher method, followed by HPLC-NMR analysis. HPLC-NMR was essential for the determination of the absolute configuration of retroflexanone (**1**) as conventional NMR methods were found to result in the degradation of the retroflexanone Mosher esters before analysis could be carried out.

## 2. Results and Discussion

**Scheme 1:**
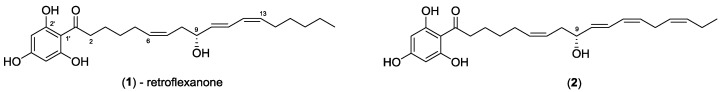
Phloroglucinols isolated from *Cystophora retroflexa.*

The dichloromethane crude extract of *C. retroflexa* was subjected to silica gel flash chromatography to yield retroflexanone (**1**), and a structurally related phloroglucinol analogue (**2**) in an enriched fraction (Scheme 1). The enriched fraction consisted of an approximate 3:1 ratio of the compounds (**2**) and (**1**) respectively. This enriched fraction was subjected to a complete 2D NMR analysis (via HPLC-NMR) whereby acquisition of the 2D NMR spectra for both compounds was achieved in the stop-flow mode. This permitted each compound to be structurally assigned. This approach would allow for rapid structure assignment of the Mosher esters based only on the ^1^H NMR comparison of the Mosher esters to the natural compound.

The enriched fraction was subjected to the advanced Mosher method using α-methoxy-α-trifluoromethylphenylacetyl chloride (MPTA-Cl). As there was a greater amount of (**2**) present in the fraction, it was suspected that this would be derivatised preferentially over retroflexanone (**1**).

The enriched fraction was reacted with dry pyridine and (*R*)-(−)-MTPA-Cl and (*S*)-(+)-MTPA-Cl (α-methoxy-α-trifluoromethylphenylacetyl chloride) respectively to prepare the Mosher esters, paying particular attention to the fact that the (*R*)-(−)-MTPA-Cl gives the (*S*)-MTPA ester and vice versa [3]. Esterification yielded the diastereoisomeric (*S*)-MTPA and (*R*)-MTPA esters. The ^1^H NMR chemical shift differences between the MTPA esters [Δδ*_SR_* = δ*_s_* − δ*_R_*] were established by comparing the ^1^H NMR spectra of the esters to that of the original ^1^H NMR spectrum of retraflexaonone, and are given in Table 1.

Each reaction was subsequently analysed individually via HPLC-NMR which revealed the presence of a new peak in the HPLC chromatogram, corresponding to the Mosher esters of the structurally related phloroglucinol analogue (**2a** and **2b**) respectively. The absolute configuration of (**2**) has previously been determined using the Horeau, and ozonolysis methods [12]. Analysis and interpretation of the ^1^H NMR data for this compound provided a means to assess whether the formation of Mosher esters formed in a mixture could unequivocally provide the correct absolute configuration. By observing which of the proton chemical shifts and therefore which substituent is affected by the formation of each derivative, the absolute configuration of the secondary alcohol is able to be established. The ^1^H NMR chemical shift differences between the MTPA esters [Δδ*^SR^* = δ*_s_* − δ*_R_*] were established for compound (**2**) (see Table 1). Despite some of the δ*^SR^* values being unresolved or solvent suppressed in the HPLC-NMR analysis, the values that could be obtained were allocated within the same substituent based on their sign (positive or negative) and this permitted the absolute configuration of (**2**) to be deduced as being *R*, which was in agreement with the previous findings (refer to the retroflexanone example for complete details of the analysis and the supporting information file). With the methodology secured, the determination of retraflexonanone (**1**) was targeted. 

It was noted during the HPLC-NMR analysis of the advanced Mosher reactions, that both retroflexanone (**1**) and the structurally related phloroglucinol (**2**) were still present in the enriched fraction, and that neither had completely reacted with the Mosher reagents. Moreover the Mosher esters of retroflexanone (**1**) were not observed. The reaction was carried out on a number of occasions using slightly different reaction conditions, but each time the Mosher esters of retroflexanone (**1**) were not observed.

**Figure 1:**
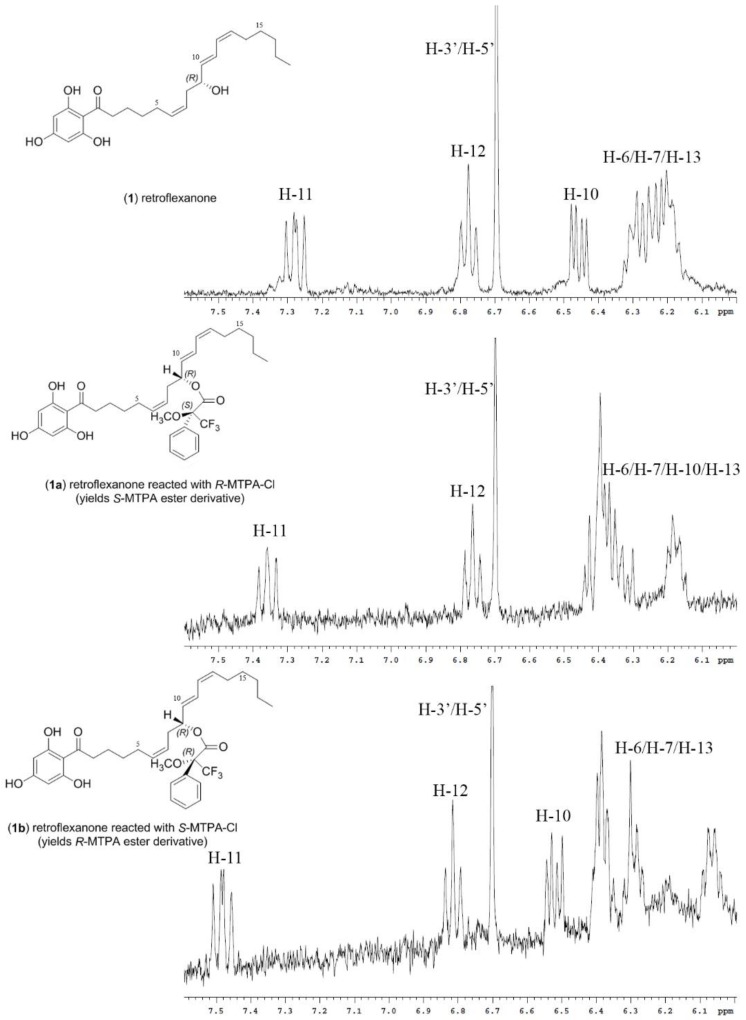
Stop-flow (HPLC-NMR) expansions of the ^1^H NMR spectra of retroflexanone (**1**) and Mosher ester derivatives (**1a** and **1b**) showing characteristic chemical shift influences.

An alternative approach was taken where retroflexanone (**1**) was rapidly purified from the enriched fraction by semi-preparative reversed phase HPLC. It was eventually established that retroflexanone (**1**) would remain intact long enough if kept in dichloromethane or methanol at −80 °C, but that it would degrade rapidly in the presence of chloroform or if kept at room temperature. Retroflexanone (**1**) was reacted with dry pyridine and the Mosher reagents (*R*)-(−)-MTPA-Cl and (*S*)-(+)-MTPA-Cl respectively. Subsequent HPLC-NMR analysis was performed on each of the reactions and this revealed the presence of the new Mosher ester derivatives of retroflexanone (**1a** and **1b**), together with residual retroflexanone (**1**) that had not reacted with the Mosher reagents. Stop-flow HPLC-NMR was carried out on the two Mosher esters (**1a** and **1b**) to obtain their corresponding ^1^H NMR spectra. The ^1^H NMR spectrum (Figure 1) of retroflexanone (**1**) was compared to the spectra obtained for the Mosher esters (**1a** and **1b**). Characteristic upfield and downfield shifts of the ^1^H NMR chemical shifts were noted depending on their occurrence on either side of the stereogenic secondary alcohol.

**Figure 2:**
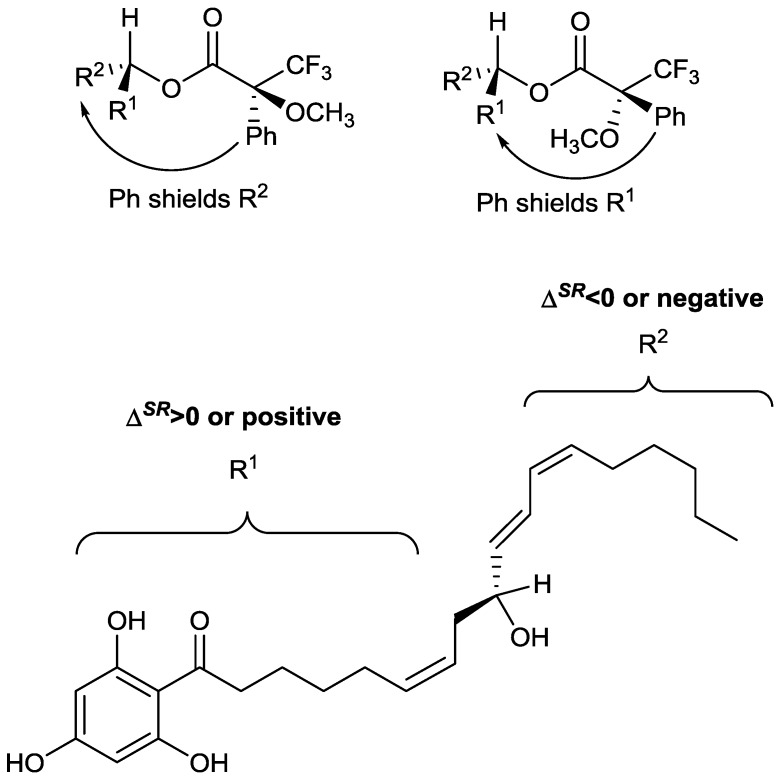
Diastereomeric MTPA esters of retroflexanone.

Differences in the proton chemical shifts of **1a** and **1b** are given in Table 1. The majority of the Δδ*^SR^* values could be calculated despite some unassigned or solvent suppressed signals. The two diastereomeric MTPA esters for retroflexanone are given in Figure 2, with R^1^ and R^2^ representing that alkyl chains on either side of the secondary alcohol moiety. It is shown in Figure 2 that the phenyl group from the MTPA will either shield the R^1^ or R^2^ group, depending on which diastereomer is present.

Using the conformations shown in Figure 2 for the MTPA esters of generic alcohols, the R^1^ and R^2^ substituents could be assigned. Protons that have positive Δδ*^SR^* values reside within R^1^, whereas those with negative values of Δδ*^SR^* belong to the R^2^ substituent [13]. Figure 2 shows that all of the positive Δδ*^SR^* are located on the left hand side, while the negative Δδ*^SR^* are located on the right hand side of the molecule. Specifically protons with positive Δδ*^SR^* values (those in R^1^) are all on one side (front) of the plane of the MTPA moiety, whereas those with negative values are all on the opposite (back) side of that plane. This, in conjunction with the Cahn Ingold Prelog convention, permitted the assignment of the original secondary alcohol absolute configuration to be assigned as *R*.

Since the structure of retroflexanone (**1**) had previously been successfully and unequivocally characterised by HPLC-NMR [1], an attempt was made to dissolve pure retroflexanone (**1**) in CDCl_3_ to obtain conventional NMR data. Despite retroflexanone (**1**) being unstable in CDCl_3_, this solvent was chosen to make the comparison to other related compounds in the literature easier. 1D and 2D NMR data was successfully obtained, however the methylene protons at position C-2 displayed unexpected splitting patterns. It was expected that these methylene protons would display a typical triplet. However the methylene at position C-2 appeared as a set of non-equivalent protons displaying a multiplet-type splitting. Initially it was thought that retroflexanone (**1**) may have degraded, but subsequent interpretation of the NMR data still supported the initial structure. It was proposed that when retroflexanone (**1**) and other structurally related compounds are dissolved in CDCl_3_, that non-equivalent protons displaying multiplet-type splitting is observed rather than a typical triplet due to a pro-chiral effect created by the carbonyl group at C-1. To test this theory, the structurally related analogue (**2**) was placed into CDCl_3_ and CD_3_OD. The NMR data revealed that in CDCl_3_ the methylene protons at position C-2 displayed multiplet-type splitting, but in CD_3_OD the methylene protons appear as a typical triplet (see supporting information section). This confirmed that the appearance of these methylene protons is dependent on the NMR solvent used for acquisition. Detailed NMR data can be found in supporting materials.

## 3. Materials and Methods

### 3.1. General Experimental Procedures

All organic solvents used were analytical reagent (AR or GR), UV spectroscopic, or HPLC grades with Milli-Q water also being used. ^1^H (500 MHz) and ^13^C (125 MHz) NMR spectra were acquired in CDCl_3_ and CD_3_OD on a 500 MHz Agilent DD2 NMR spectrometer, or a 300 MHz Bruker Avance III NMR spectrometer with referencing to solvent signals (δ_H_ 7.26; δ_C_ 77.0 and, δ_H_ 3.31; δ_C_ 49.0). Two-dimensional NMR experiments recorded included gradient correlation spectroscopy (gCOSY), heteronuclear single-quantum correlation spectroscopy with adiabatic pulses (HSQCAD), and gradient heteronuclear multiple-bond spectroscopy with adiabatic pulses (gHMBCAD).

### 3.2. Alga Material

*C. retroflexa* was collected via SCUBA on 21 April 2010 from Governor Reef (near Indented Head), Port Phillip Bay, Victoria, Australia. The alga was identified by Dr Gerry Kraft (The University of Melbourne) and a voucher specimen (designated the code number 2010-09) is deposited at the School of Science (Applied Chemistry and Environmental Science), RMIT University.

### 3.3. Fractionation of Dichloromethane Crude Extract

The alga (133 g, wet weight) was extracted with 3:1 MeOH/CH_2_Cl_2_ (1 L). The crude extract was decanted and concentrated under reduced pressure and sequentially solvent partitioned (triturated) into CH_2_Cl_2_ and MeOH soluble extracts, respectively. A portion of the CH_2_Cl_2_ crude extract (445 mg) of *C. retroflexa* was subjected to silica gel flash chromatography. Silica gel flash chromatography was carried out using Davisil LC35Å silica gel (40–60 mesh) using a 20% stepwise solvent elution from 100% petroleum spirits (60–80 °C) to 100% CH_2_Cl_2_ to 100% EtOAc and finally to 100% MeOH. The 60% CH_2_Cl_2_/EtOAc fraction yielded a mixture which contained retroflexanone (**1**) and the structurally related phloroglucinol (**2**) (136.2 mg).

### 3.4. Advanced Mosher Derivatisation of Phloroglucinol *(**2**)* in an Enriched Fraction

The 60% CH_2_Cl_2_/EtOAc fraction (1 mg) was dissolved in CH_2_Cl_2_ (1 mL) to which (*R*)-(−)-MTPA-Cl (4.0 mg, 16 µmol) or alternatively (*S*)-(+)-MTPA-Cl (4.5 mg, 18 µmol) was added. Dry pyridine (200 µL, 760 µmol) was then added and the reaction stirred for 4 h. The reaction mixtures were then dried under a stream of nitrogen and reconstituted into HPLC-NMR grade CH_3_CN.

### 3.5. HPLC Purification of Retroflexanone

Semi-preparative HPLC was carried out on a Dionex P680 (solvent delivery module) equipped with a Dionex UVD340U PDA detector and a Foxy Jr. automated fraction collector. An isocratic HPLC method (85% CH_3_CN/H_2_O) was employed using a Phenomenex Luna (2) 100 Å C18 250 × 10 mm (5 µm) HPLC column. The automatic fraction collector was programmed to collect the compounds based on their retention times at a flow rate of 4.0 mL/min. A portion of the 60% CH_2_Cl_2_/EtOAc fraction was subjected to reversed phase HPLC to yield retroflexanone (**1**) (2.8 mg) and the phloroglucinol (**2**) (6.0 mg).

### 3.6. Advanced Mosher Reaction of Retroflexanone

Retroflexanone (1.4 mg, 3.5 µmol) was dissolved in CH_2_Cl_2_ (1 mL) to which (*R*)-(−)-MTPA-Cl (50 mg, 198 µmol, 57 equiv.) or alternatively (*S*)-(+)-MTPA-Cl (50 mg, 198 µmol, 57 equiv.) was added. Dry pyridine (300 µL, 1140 µmol, 325 equiv.) was then added and the reaction stirred for 4 h. The reaction mixtures were then dried under a stream of nitrogen and reconstituted into HPLC-NMR grade CH_3_CN.

### 3.7. HPLC-NMR Analysis

For HPLC-NMR details, please refer to Brkljaca and Urban [14]. For stop-flow HPLC-NMR modes, 50-μL injections of the reconstituted samples in CH_3_CN were injected onto an Agilent Eclipse Plus C_18_ (150 × 4.6) 5-µ column using a solvent composition of 75% CH_3_CN/D_2_O at a flow rate of 1 mL/min. In the stop-flow HPLC-NMR mode WET1D NMR experiments were acquired.

### 3.8. On-Line NMR Data Obtained via HPLC-NMR

*Retroflexanone or 9R-hydroxy-1-(2,4,6-trihydroxyphenyl)-6Z,10E,12Z-Octadecatrien-1-one reacted with (R)-(−)-MTPA-Cl yielding the S-MTPA ester* (**1a**); ^1^H NMR (500 MHz, 75% CH_3_CN/D_2_O) δ 8.28 (5H, m, MTPA-aromatic), 7.36 (1H, dd, *J* = 11.5, 13.0 Hz, H-11), 6.76 (1H, dd, *J* = 11.0, 11.5 Hz, H-12), 6.70 (2H, s, H-3′/H-5′), 6.14−6.45 (4H, m, H-6/H-7/H-10/H-13), 4.83 (1H, m, H-9), 4.35 (3H, s, MTPA-OCH_3_), 3.85 (2H, t, *J* = 7.5 Hz, H-2), 3.33 (2H, m, H-8), 2.44 (2H, m, H-3), 2.18 (2H, m, H-4), 2.10 (2H, m, H-17), 1.70 (3H, t, *J* = 7.0 Hz, H-18), ND (11H, H-5/H-14/H-15/H-16,/2′-OH/4′-OH/6′-OH), ND indicates signal not detected.

*Retroflexanone or 9R-hydroxy-1-(2,4,6-trihydroxyphenyl)-6Z,10E,12Z-Octadecatrien-1-one reacted with (S)-(+)-MTPA-Cl yielding the R-MTPA ester* (**1b**); ^1^H NMR (500 MHz, 75% CH_3_CN/D_2_O) δ 8.28 (5H, m, MTPA-aromatic), 7.48 (1H, dd, *J* = 11.0, 15.5 Hz, H-11), 6.82 (1H, dd, *J* = 10.5, 11.5 Hz, H-12), 6.70 (2H, s, H-3′/H-5′), 6.52 (1H, dd, *J* = 7.5, 15.5, H-10), 6.02–6.42 (3H, m, H-6/H-7/H-13), 4.83 (1H, m, H-9), 4.32 (3H, s, MTPA-OCH_3_), 3.84 (2H, t, *J* = 7.5 Hz, H-2), 3.26 (2H, m, H-8), 2.43 (2H, m, H-3), 2.16 (2H, m, H-4), 2.10 (2H, m, H-17), 1.70 (3H, m, H-18), ND (11H, H-5/H-14/H-15/H-16/2′-OH/4′-OH/6′-OH), ND indicates signal not detected.

*9R-hydroxy-1-(2,4,6-trihydroxy-phenyl)-6Z,10E,12Z,15Z-Octadecatetraen-1-one reacted with (R)-(−)-MTPA-Cl yielding the S-MTPA ester* (**2a**); ^1^H NMR (500 MHz, 75% CH_3_CN/D_2_O) δ 8.27 (5H, m, MTPA-aromatic), 7.38 (1H, dd, *J* = 11.5, 14.5 Hz, H-11), 6.76 (1H, dd, *J* = 10.5, 11.5 Hz, H-12), 6.69 (2H, s, H-3′/H-5′), 6.07−6.46 (6H, m, H-6/H-7/H-10/H-13/H-15/H-16), 4.34 (3H, s, MTPA-OCH_3_), 3.84 (2H, t, *J* = 7.5 Hz, H-2), 3.65 (2H, m, H-14), 3.35 (1H, m, H-8a), 3.29 (1H, m, H-8b), 2.42 (2H, p, *J* = 7.5 Hz, H-3), 2.19 (2H, p, *J* = 7.5 Hz, H-4), 1.75 (3H, t, *J* = 7.5 Hz, H-18), ND (8H, H-5/H-9/H-17/2′-OH/4′-OH/6′-OH), ND indicates signal not detected.

*9R-hydroxy-1-(2,4,6-trihydroxy-phenyl)-6Z,10E,12Z,15Z-Octadecatetraen-1-one reacted with (S)-(+)-MTPA-Cl to yield the R-MTPA ester* (**2b**); ^1^H NMR (500 MHz, 75% CH_3_CN/D_2_O) δ 8.30 (5H, m, MTPA-aromatic), 7.52 (1H, dd, *J* = 11.0, 15.5 Hz, H-11), 6.82 (1H, dd, *J* = 10.5, 11.0 Hz, H-12), 6.70 (2H, s, H-3′/H-5′), 6.55 (1H, dd, *J* = 7.5, 15.5 Hz, H-10), 6.04−6.42 (5H, m, H-6/H-7/H-13/H-15/H-16), 4.32 (3H, s, MTPA-OCH_3_), 3.84 (2H, t, *J* = 7.0 Hz, H-2), 3.73 (2H, m, H-14), 3.31 (1H, m, H-8a), 3.24 (1H, m, H-8b), 2.41 (2H, p, *J* = 7.0 Hz, H-3), 2.15 (2H, p, *J* = 7.0 Hz, H-4), 1.76 (3H, t, *J* = 7.0 Hz, H-18), ND (8H, H-5/H-9/H-17/2′-OH/4′-OH/6′-OH), ND indicates signal not detected.

### 3.9. Off-Line NMR Data

*Retroflexanone* (**1**); unstable oil that darkened over time; ^1^H NMR (500 MHz, CDCl_3_) δ 6.52 (1H, dd, *J* = 11.5, 15.0 Hz, H-11), 5.97 (1H, dd, *J* = 11.0, 11.5 Hz, H-12), 5.88 (2H, s, H-3′/H-5′), 5.70 (1H, dd, *J* = 6.0, 15.0 Hz, H-10), 5.54 (1H, m, H-6), 5.45 (1H, dt, *J* = 7.0, 11.0 Hz, H-13), 5.39 (1H, m, H-7), 4.26 (1H, dt, *J* = 6.0, 6.5 Hz, H-9), 3.14 (1H, m, H-2a), 3.00 (1H, m, H-2b), 2.41 (1H, m, H-8a), 2.31 (1H, m, H-8b), 2.17 (2H, m, H-14), 2.12 (2H, m, H-5), 1.72 (2H, m, H-3), 1.48 (2H, m, H-4), 1.37 (2H, m, H-15), 1.29 (4H, m, H-16/H-17), 0.88 (3H, t, *J* = 7.0 Hz, H-18), ND (9-OH/2′-OH/4′-OH/6′-OH), ND indicates signal not detected; ^13^C NMR (125 MHz, CDCl3_3_) δ 134.6 (CH, C-10), 133.5 (CH, C-6), 133.4 (CH, C-13), 127.5 (CH, C-12), 126.1 (CH, C-11), 124.7 (CH, C-7), 95.4 (CH, C-3′/C-5′), 72.5 (CH, C-9), 43.6 (CH_2_, C-2), 35.2 (CH_2_, C-8), 31.4 (CH_2_, C-16), 29.3 (CH_2_, C-4/C-15), 27.4 (CH_2_, C-14), 26.8 (CH_2_, C-5), 24.4 (CH_2_, C-3), 22.6 (CH_2_, C-17), 14.1 (CH_3_, C-18), ND (C-1/C-1′/C-2′/C-4′/C-6′), ND indicates signal not detected.

*9R-hydroxy-1-(2,4,6-trihydroxy-phenyl)-6Z,10E,12Z,15Z-Octadecatetraen-1-one* (**2**); unstable oil that darkened over time; ^1^H NMR (500 MHz, CDCl_3_) δ 6.55 (1H, dd, *J* = 11.5, 15.0 Hz, H-11), 5.98 (1H, dd, *J* = 11.0, 11.0 Hz, H-12), 5.88 (2H, bs, H-3′/H-5′), 5.72 (1H, dd, *J* = 6.0, 14.5 Hz, H-10), 5.27−5.59 (5H, m, H-6/H-7/H-13/H-15/H-16), 4.27 (1H, dt, *J* = 6.0, 6.5 Hz, H-9), 3.11 (1H, m, H-2a), 3.00 (1H, m, H-2b), 2.93 (2H, dd, *J* = 7.0, 7.5 Hz, H-14), 2.39 (1H, m, H-8a), 2.32 (1H, m, H-8b), 2.12 (4H, m, H-5/H-17), 1.71 (2H, m, H-3), 1.48 (2H, p, *J* = 7.0 Hz, H-4), 0.97 (3H, t, *J* = 7.5 Hz, H-18), ND (9-OH/2′-OH’4′-OH/6′-OH), ND indicates signal not detected; ^1^H NMR (300 MHz, CD_3_OD) δ 6.55 (1H, dd, *J* = 11.1, 15.0 Hz, H-11), 5.97 (1H, dd, *J* = 10.8, 11.1 Hz, H-12), 5.83 (2H, s, H-3′/H-5′), 5.68 (1H, dd, *J* = 6.6, 15.0 Hz, H-10), 5.27−5.55 (5H, m, H-6/H-7/H-13/H-15/H-16), 4.15 (1H, dt, *J* = 6.3, 6.6 Hz, H-9), 3.06 (2H, t, *J* = 7.5 Hz, H-2), 2.94 (2H, dd, *J* = 7.0, 7.5 Hz, H-14), 2.32 (2H, m, H-8), 2.10 (4H, m, H-5/H-17), 1.69 (2H, p, *J* = 7.5 Hz, H-3), 1.45 (2H, p, *J* = 7.5 Hz, H-4), 0.98 (3H, t, *J* = 7.5 Hz, H-18), ND (9-OH/2′-OH’4′-OH/6′-OH), ND indicates signal not detected.

## 4. Conclusions

HPLC-NMR in conjunction with the advanced Mosher method was successful in confirming the absolute configuration of two related phloroglucinol compounds. The absolute configuration of retroflexanone (**1**) was achieved via off-line isolation followed by HPLC-NMR analysis, whilst compound (**2**) was secured via the advanced Mosher method HPLC-NMR analysis of an enriched fraction. Both compounds displayed an *R* absolute configuration at the secondary alcohol. This represents, to the best of our knowledge, the first instance of the modified Mosher method combined with HPLC-NMR being employed on an enriched fraction rather than individual purified compounds. 

## Figures and Tables

**Table 1:** ^1^H NMR data of compounds (**1**) and (**2**) and the characteristic chemical shift value differences Δδ*^SR^* of the corresponding Mosher esters, recorded at 500 MHz (75% CH_3_CN/D_2_O).

**Position**	**δ_H_ Retroflexanone (1) (ppm)**	**δ_H_ Retroflexanone Reacted with *R*-MTPA-Cl to Give *S*-MTPA Ester (1a) (ppm)**	**δ_H_ Retroflexanone Reacted with *S*-MTPA-Cl to Give *R*-MTPA Ester (1b) (ppm)**	**Δδ*^SR^* (ppm)**	**Δδ*^SR^* (Hz)**
2	3.86, t (7.0)	3.85, t (7.5)	3.84, t (7.5)	+0.01	+6
3	2.44, p (7.0)	2.44, m	2.43, m	+0.01	+7
4	2.20, m	2.18, m	2.16, m	+0.02	+9
8a	3.09, m	3.33, m	3.26, m	+0.07	+42
8b	3.09, m	3.33, m	3.26, m	+0.07	+42
10	6.45, dd (6.5, 15.0)	6.14–6.45, m	6.52, dd (7.5, 15.5)	(negative value) ^a^	(negative value) ^a^
11	7.28, dd (11.0, 15.0)	7.36, dd (11.5, 13.0)	7.48, dd (11.0, 15.5)	−0.12	−62
12	6.78, dd (10.5, 10.5	6.76, dd (11.0, 11.5)	6.82, dd (10.5, 11.5)	−0.16	−26
14	3.09, m	ND	ND	n.a.	n.a.
18	1.70, t (7.0)	1.70, t (7.0)	1.70, m	0	0
**Position**	**δ_H_ Phloroglucinol (2) (ppm)**	**δ_H_ Phloroglucinol Reacted with *R*-MTPA-Cl to Give *S*-MTPA Ester (2a) (ppm)**	**δ_H_ Phloroglucinol Reacted with *S*-MTPA-Cl to Give *R*-MTPA ester (2b) (ppm)**	**Δδ*^SR^* (ppm)**	**Δδ*^SR^* (Hz)**
2	3.86, t (7.0)	3.84, t (7.5)	3.84, t (7.0)	0	0
3	2.45, p (7.0)	2.42, p (7.5)	2.41, p (7.0)	+0.01	+9
4	2.22, p (7.0)	2.19, p (7.5)	2.15, p (7.0)	+0.04	+20
8a	3.10, m	3.35, m	3.31, m	+0.04	+30
8b	3.10, m	3.29, m	3.24, m	+0.05	+31
10	6.47, dd (6.5, 15.0)	6.07–6.46, m	6.55, dd (7.5, 15.5)	(negative value) ^a^	(negative value) ^a^
11	7.32, dd (11.0, 15.0)	7.38, dd (11.5, 14.5)	7.52, dd (11.0, 15.5)	−0.14	−70
12	6.79, dd (11.0, 11.0)	6.76, dd (10.5, 11.5)	6.82, dd (10.5, 11.0)	−0.06	−30
14	3.74, dd (7.0, 14.5)	3.65, m	3.73, m	−0.08	−38
18	1.77, t (7.0)	1.75, t (7.5)	1.76, t (7.0)	−0.01	−3

ND—indicates signal was not detected due to signal suppression; n.a. Δδ*^SR^* not available due to signal overlap or signal suppression; ^a^ exact Δδ*^SR^* could not be determined due to signal overlap, but it was evident that the Δδ*^SR^* value was negative.

## Supplementary Materials

The following are available online at http://www.mdpi.com/1660-3397/16/6/205/s1, supplementary materials S1–S12: NMR data of all compounds analyzed in this article.
