# Supplementary material for: Absolute Configuration Determination of Retroflexanone Using the Advanced Mosher Method and Application of HPLC-NMR
Source: Mar Drugs. 2018 Jun 12;16(6):205. doi: 10.3390/md16060205 (PMC6025468; doi:10.3390/md16060205)
Supplement: Supplementary file 1 [file marinedrugs-16-00205-s001.pdf]

Absolute configuration determination of retroflexanone by the advanced Mosher Method and application of HPLC-NMR

Caleb Singleton[a], Robert Brkljača[a] and Sylvia Urban[a*]

[a]School of Science (Applied Chemistry and Environmental Science), RMIT University, GPO Box 2476 Melbourne, Victoria, 3001, Australia

Supporting Information

S1. Stop-flow WET1D Proton NMR spectrum (500 MHz, 75% CH_3CN/D_2O) of retroflexanone reacted with (R)-(-)-MTPA-Cl yielding the S-MTPA ester (**1a**).

S2. Stop-flow WET1D Proton NMR spectrum (500 MHz, 75% CH_3CN/D_2O) of retroflexanone reacted with (S)-(+)-MTPA-Cl yielding the R-MTPA ester (**1b**).

S3. Table of NMR data of **1a** and **1b**.

S4. Stop-flow WET1D Proton NMR spectrum (500 MHz, 75% CH_3CN/D_2O) of 9R-hydroxy-1-(2,4,6-trihydroxy-phenyl)-6Z,10E,12Z,15Z-Octadecatetraen-1-one reacted with (R)-(-)-MTPA-Cl yielding the S-MTPA ester (**2a**).

S5. Stop-flow WET1D Proton NMR spectrum (500 MHz, 75% CH_3CN/D_2O) of 9R-hydroxy-1-(2,4,6-trihydroxy-phenyl)-6Z,10E,12Z,15Z-Octadecatetraen-1-one reacted with (S)-(+)-MTPA-Cl yielding the R-MTPA ester (**2b**).

S6. Table of **2a** and **2b**.

S7. ^{1}H NMR spectrum (500 MHz, $CDCl_3$) of retroflexanone (**1**).

S8. gCOSY NMR spectrum (500 MHz, $CDCl_3$) of retroflexanone (**1**).

S9. HSQCAD NMR spectrum (500 MHz, $CDCl_3$) of retroflexanone (**1**).

S10. gHMBCAD NMR spectrum (500 MHz, $CDCl_3$) of retroflexanone (**1**).

S11. Table of NMR data of retroflexanone (**1**).

S12. Comparison of the upfield region of the ^{1}H NMR spectra of **2** in $CDCl_3$ (**top**) and CD_3OD (**bottom**).

*Corresponding author. Tel: +61 3 9925 3376; Fax: +61 3 9925 3747
E-mail address: sylvia.urban@rmit.edu.au (S. Urban).

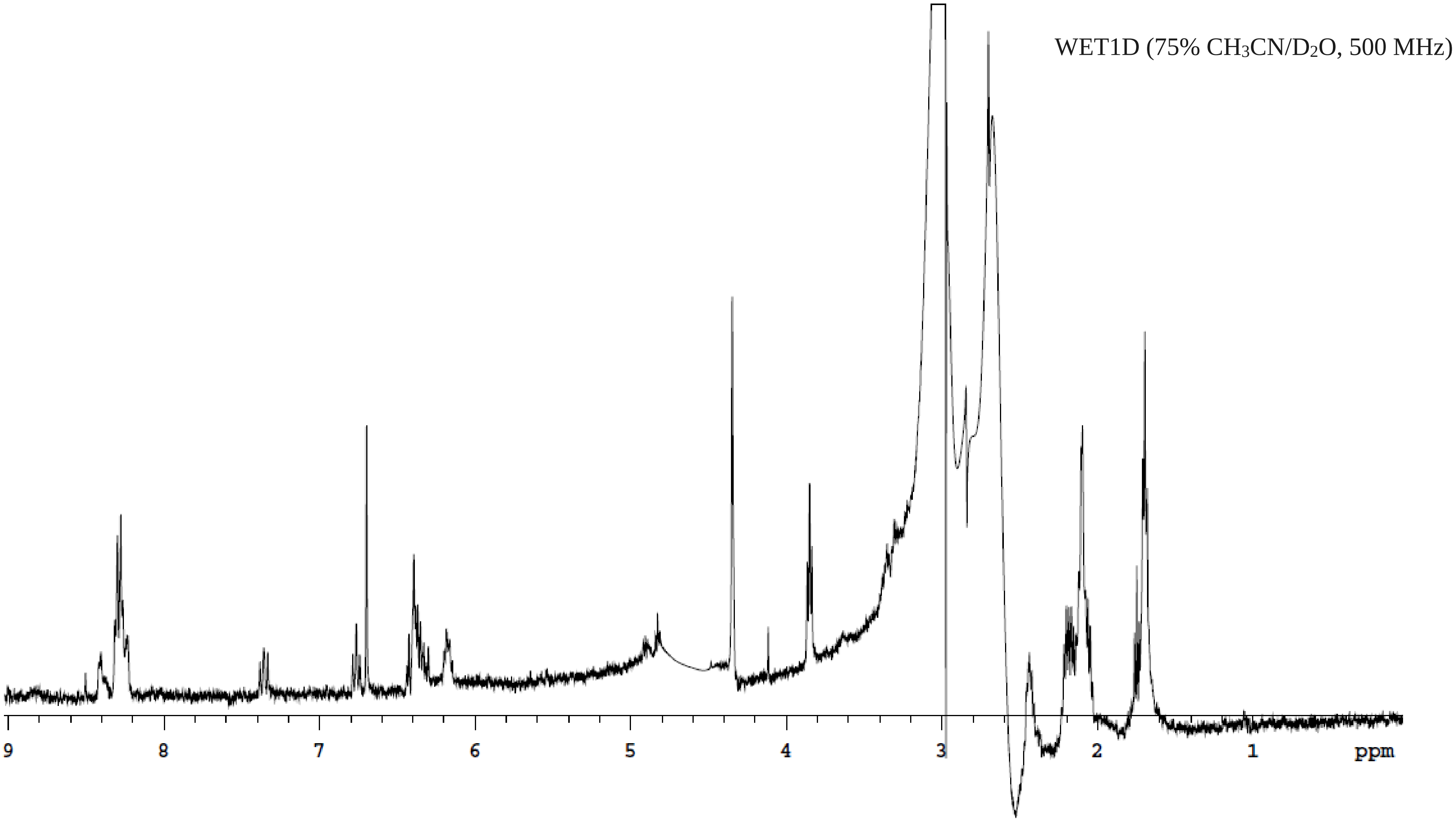

S1. Stop-flow WET1D Proton NMR spectrum (500 MHz, 75% CH_3CN/D_2O) of retroflexanone reacted with (R)-(-)-MTPA-Cl yielding the S-MTPA ester (**1a**).

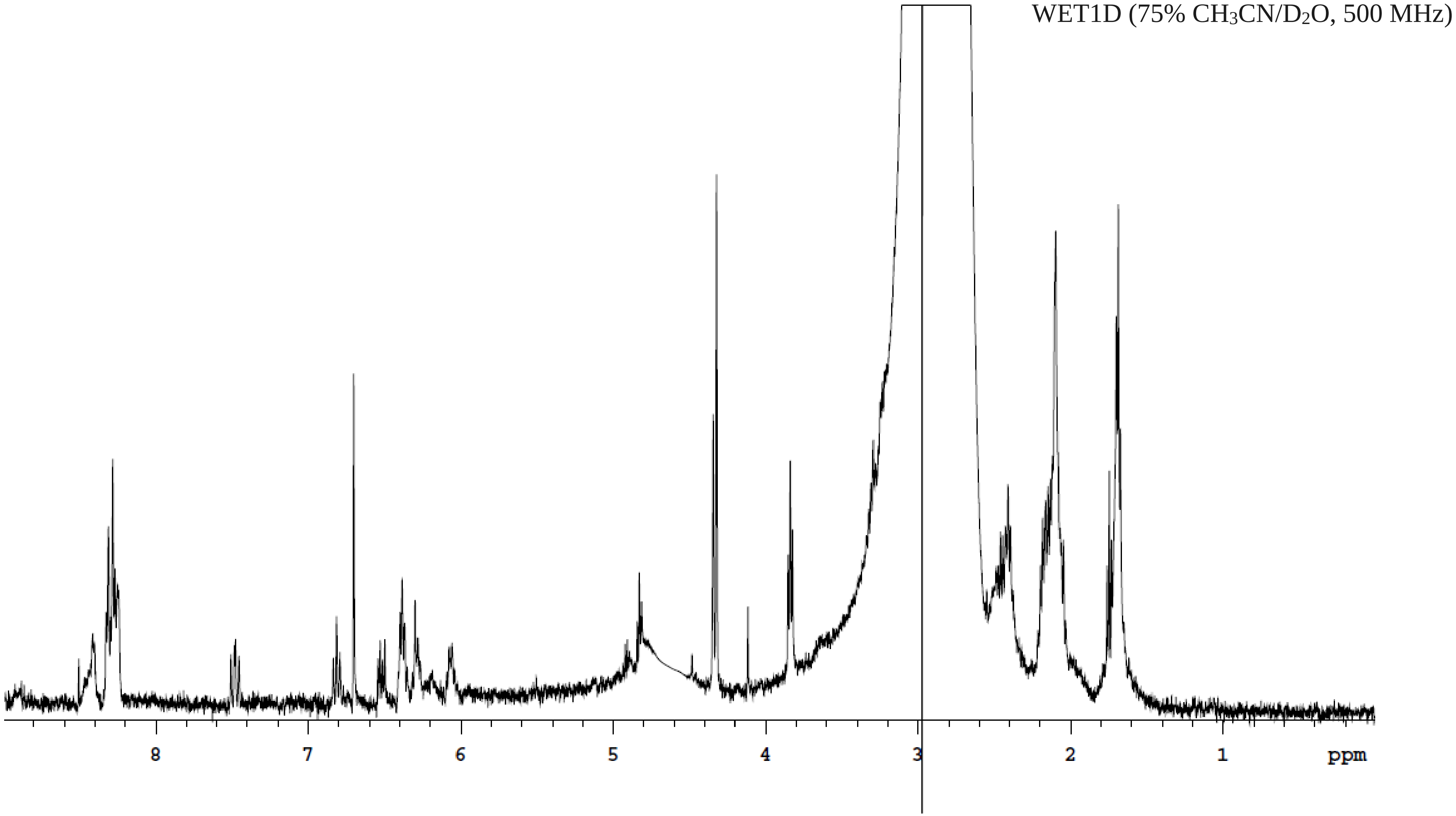

S2. Stop-flow WET1D Proton NMR spectrum (500 MHz, 75% CH_3CN/D_2O) of retroflexanone reacted with (S)-(+)-MTPA-Cl yielding the R-MTPA ester (**1b**).

S3. Table of NMR data of **1a** and **1b**.

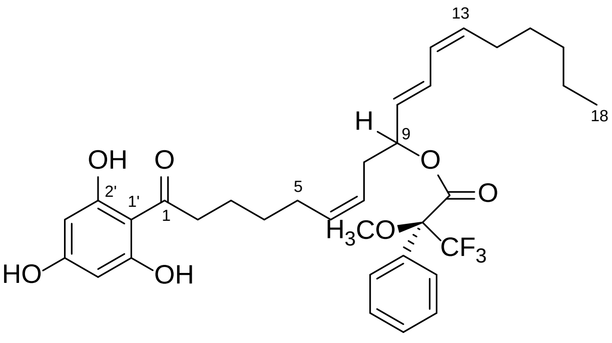

(**1a**) retroflexanone reacted with *R*-MTPA-Cl
(yields *S*-MTPA ester derivative)

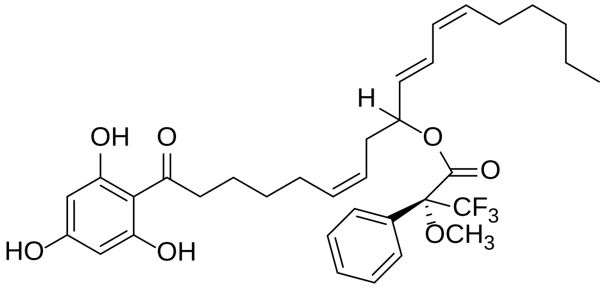

(**1b**) retroflexanone reacted with *S*-MTPA-Cl
(yields *R*-MTPA ester derivative)

**Position**	**(1a)**	**(1b)**	**$\Delta\delta^{SR}$ (ppm)**	**$\Delta\delta^{SR}$ (Hz)**
1				
2	3.85, t (7.5)	3.84, t (7.5)	+0.01	+6
3	2.44, m	2.43, m	+0.01	+7
4	2.18, m	2.16, m	+0.02	+9
5	ND	ND		
6	6.14-6.45, m	6.02-6.42, m		
7	6.14-6.45, m	6.02-6.42, m		
8	3.33, m	3.26, m	+0.07	+42
9	4.83, m	4.83, m		
10	6.14-6.45, m	6.52, dd (7.5, 15.5)	(negative value)	(negative value)
11	7.36, dd (11.5, 13.0)	7.48, dd (11.0, 15.5)	-0.12	-62
12	6.76, dd (11.0, 11.5)	6.82, dd (10.5, 11.5)	-0.16	-26
13	6.14-6.45, m	6.02-6.42, m		
14	ND	ND		
15	ND	ND		
16	ND	ND		
17	2.10, m	2.10, m	0	0
18	1.70, t (7.0)	1.70, m	0	0
1’				
2’				
3’	6.70, s	6.70, s	0	0
4’				
5’	6.70, s	6.70, s	0	0
6’				
MTPA-aromatic	8.28, m	8.28, m		
MTPA-OCH_3	4.35, s	4.32, s		
2’-OH	ND	ND		
4’-OH	ND	ND		
6’-OH	ND	ND		

Referenced to HDO (δ_H 4.64, 500 MHz
ND indicates signal not detected

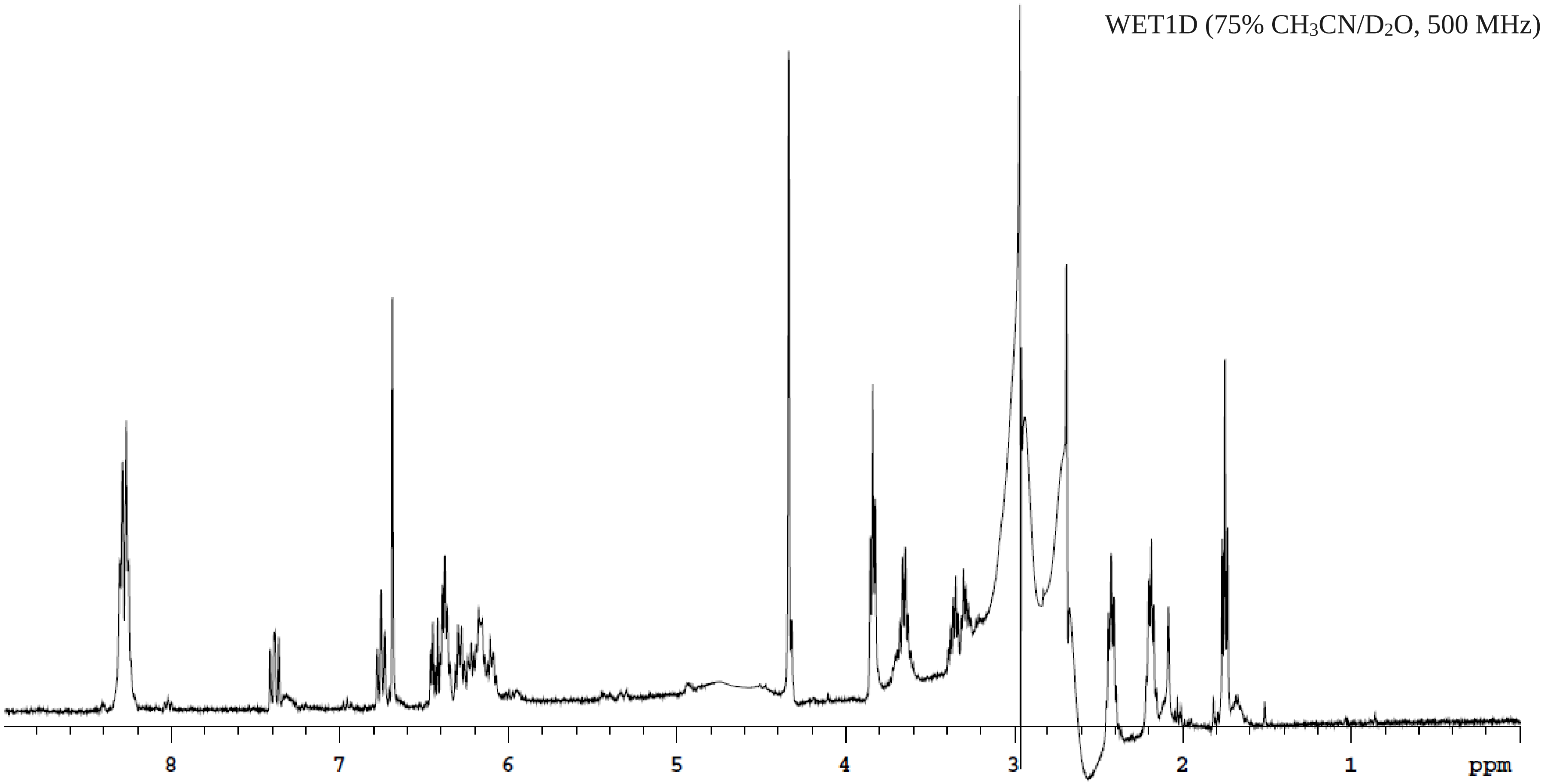

S4. Stop-flow WET1D Proton NMR spectrum (500 MHz, 75% CH_3CN/D_2O) of 9R-hydroxy-1-(2,4,6-trihydroxy-phenyl)-6Z,10E,12Z,15Z-Octadecatetraen-1-one reacted with (R)-(-)-MTPA-Cl yielding the S-MTPA ester (**2a**).

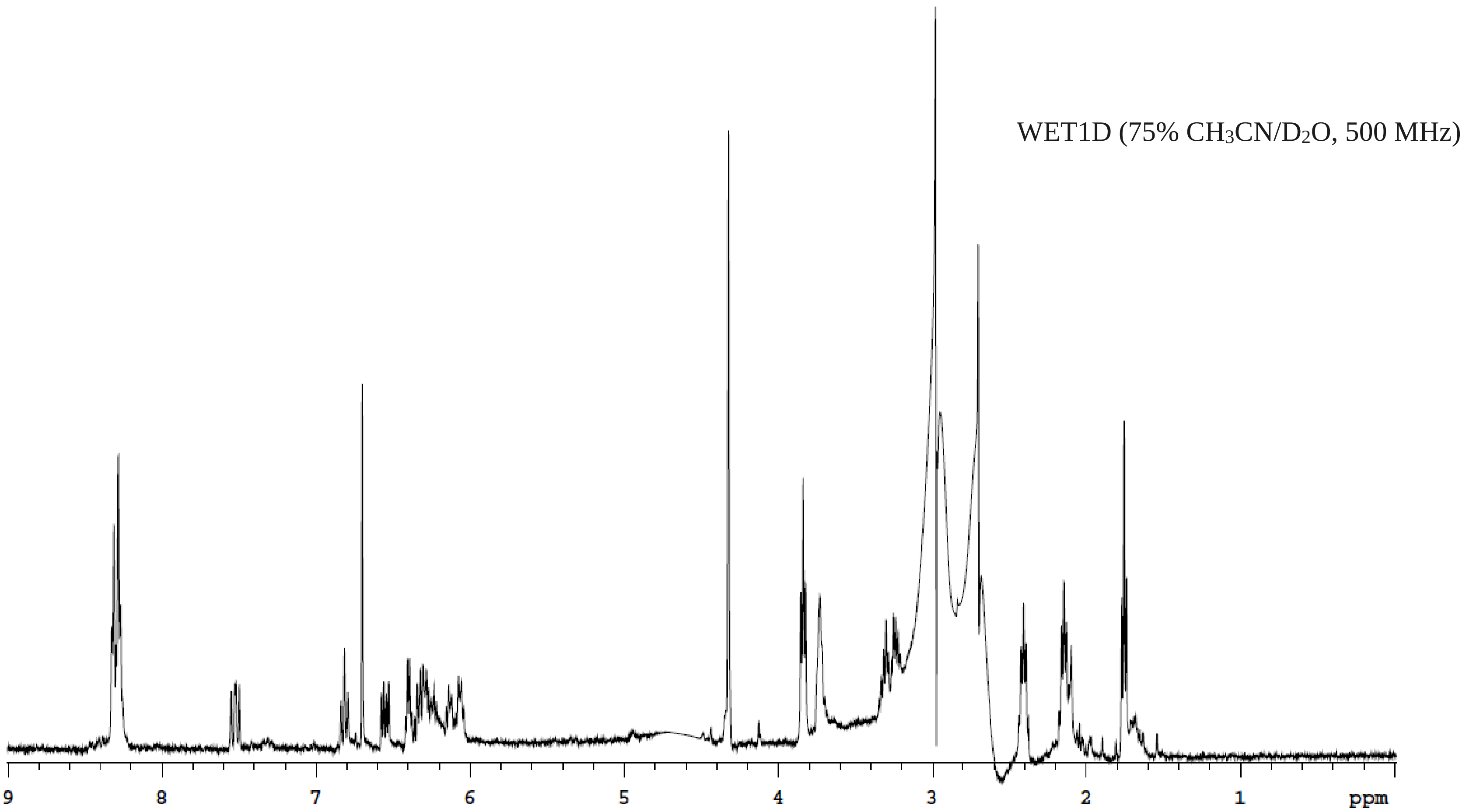

S5. Stop-flow WET1D Proton NMR spectrum (500 MHz, 75% CH_3CN/D_2O) of 9R-hydroxy-1-(2,4,6-trihydroxy-phenyl)-6Z,10E,12Z,15Z-Octadecatetraen-1-one reacted with (S)-(+)-MTPA-Cl yielding the R-MTPA ester (**2b**).

S6. Table of NMR data of **2a** and **2b**.

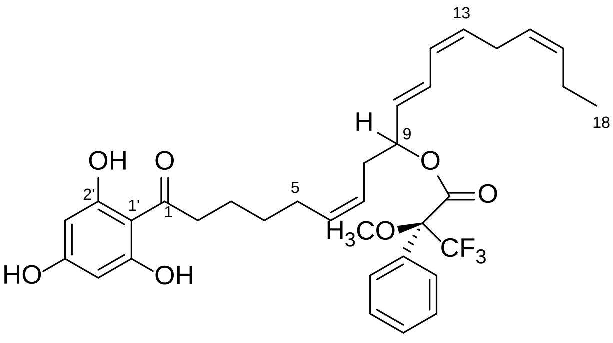

(2**a**) analogue reacted with *R*-MTPA-Cl
(yields *S*-MTPA ester derivative)

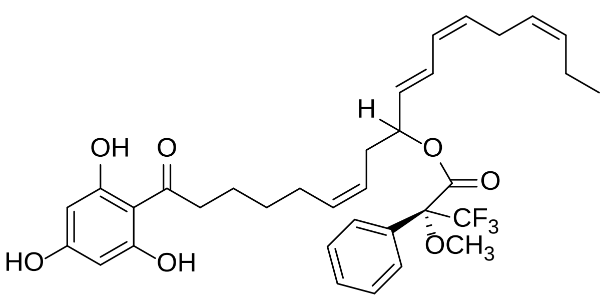

(**2b**) analogue with *S*-MTPA-Cl
(yields *R*-MTPA ester derivative)

Position	2a	2b	$\Delta\delta^{SR}$ (ppm)	$\Delta\delta^{SR}$ (Hz)
1				
2	3.84, t (7.5)	3.84, t (7.0)	0	
3	2.42, p (7.5)	2.41, p (7.0)	+0.01	+9
4	2.19, p (7.5)	2.15, p (7.0)	+0.04	+20
5	ND	ND		
6	6.07-6.46, m	6.04-6.42, m		
7	6.07-6.46, m	6.04-6.42, m		
8a	3.35, m	3.31, m	+0.04	+30
8b	3.29, m	3.24, m	+0.05	+31
9	ND	ND		
10	6.07-6.46, m	6.55, dd (7.5, 15.5)	(negative value)	(negative value)
11	7.38, dd (11.5, 14.5)	7.52, dd (11.0, 15.5)	-0.14	-70
12	6.76, dd (10.5, 11.5)	6.82, dd (10.5, 11.0)	-0.06	-30
13	6.07-6.46, m	6.04-6.42, m		
14	3.65, m	3.73, m	-0.08	-38
15	6.07-6.46, m	6.04-6.42, m		
16	6.07-6.46, m	6.04-6.42, m		
17	ND	ND		
18	1.75, t (7.5)	1.76,t (7.0)	-0.01	-3
1’				
2’				
3’	6.69, s	6.70, s	-0.01	-3
4’				
5’	6.69, s	6.70, s	-0.01	-3
6’				
MTPA-aromatic	8.27, m	8.30, m		
MTPA-OCH_3	4.34, s	4.32, s		
2’-OH	ND	ND		
4’-OH	ND	ND		
6’-OH	ND	ND		

Referenced to HDO (δ_H 4.64, 500 MHz)
ND indicates signal not detected

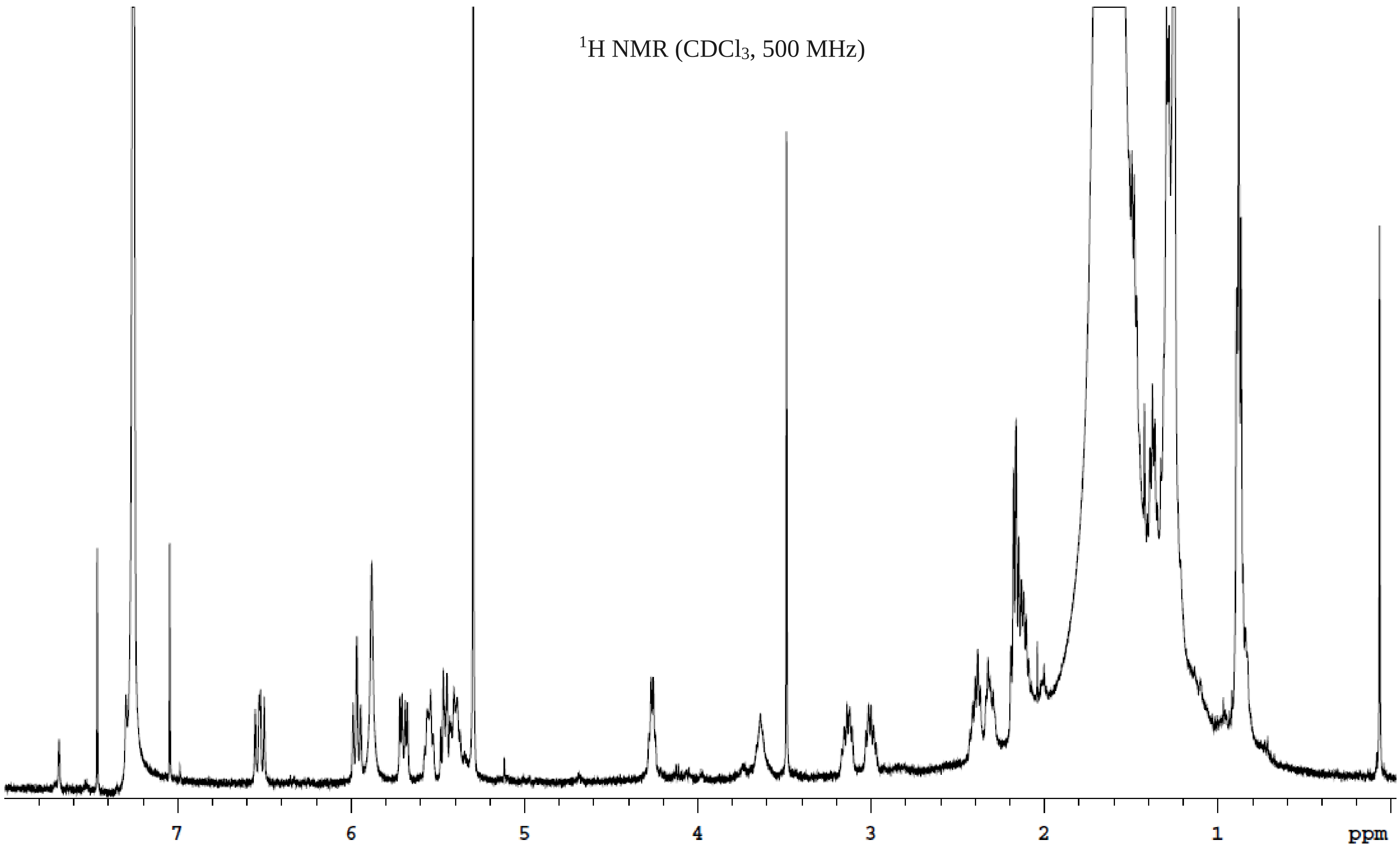

S7. ^1H NMR spectrum (500 MHz, $CDCl_3$) of retroflexanone (**1**).

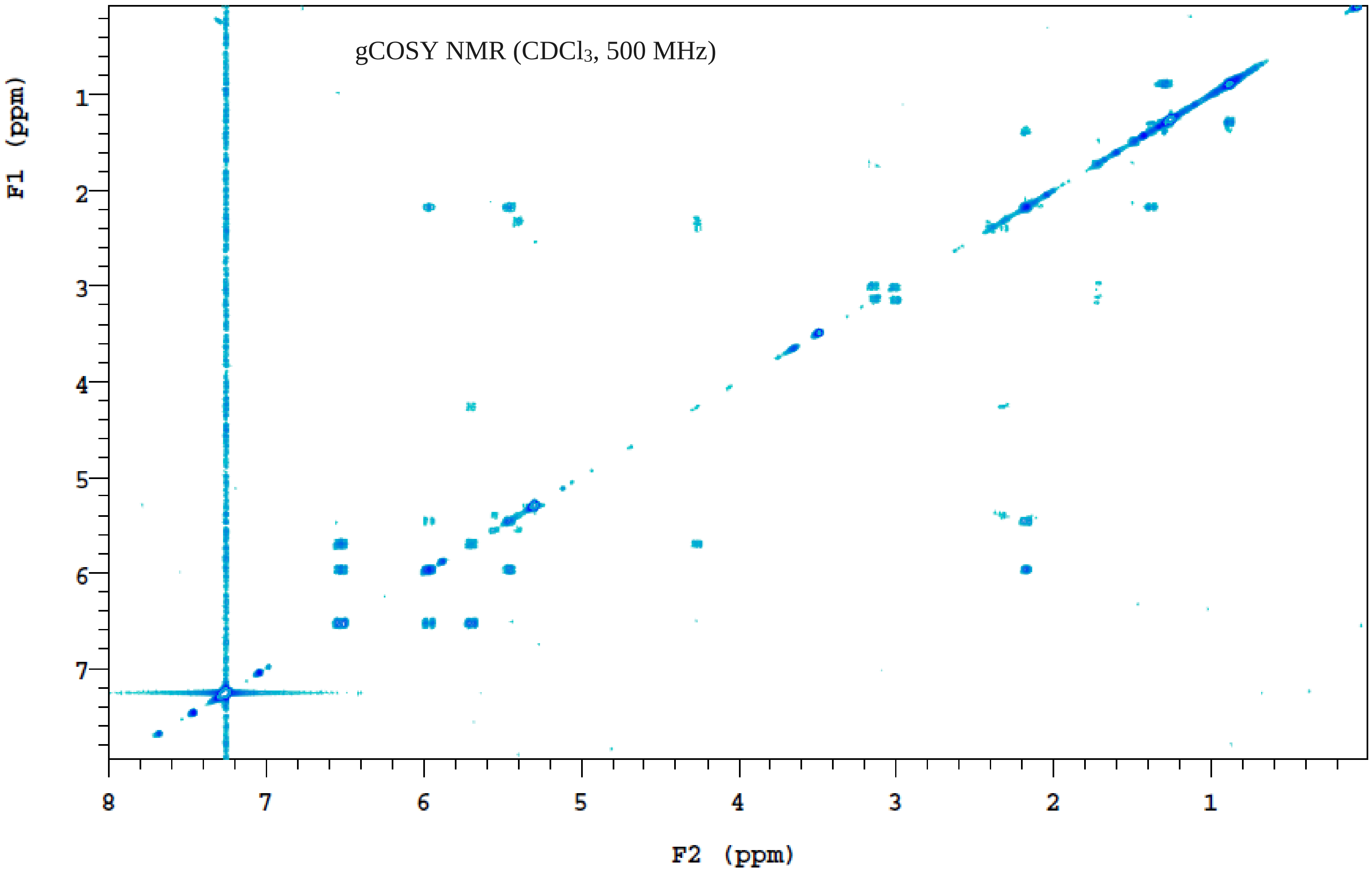

S8. gCOSY NMR spectrum (500 MHz, $CDCl_3$) of retroflexanone (**1**).

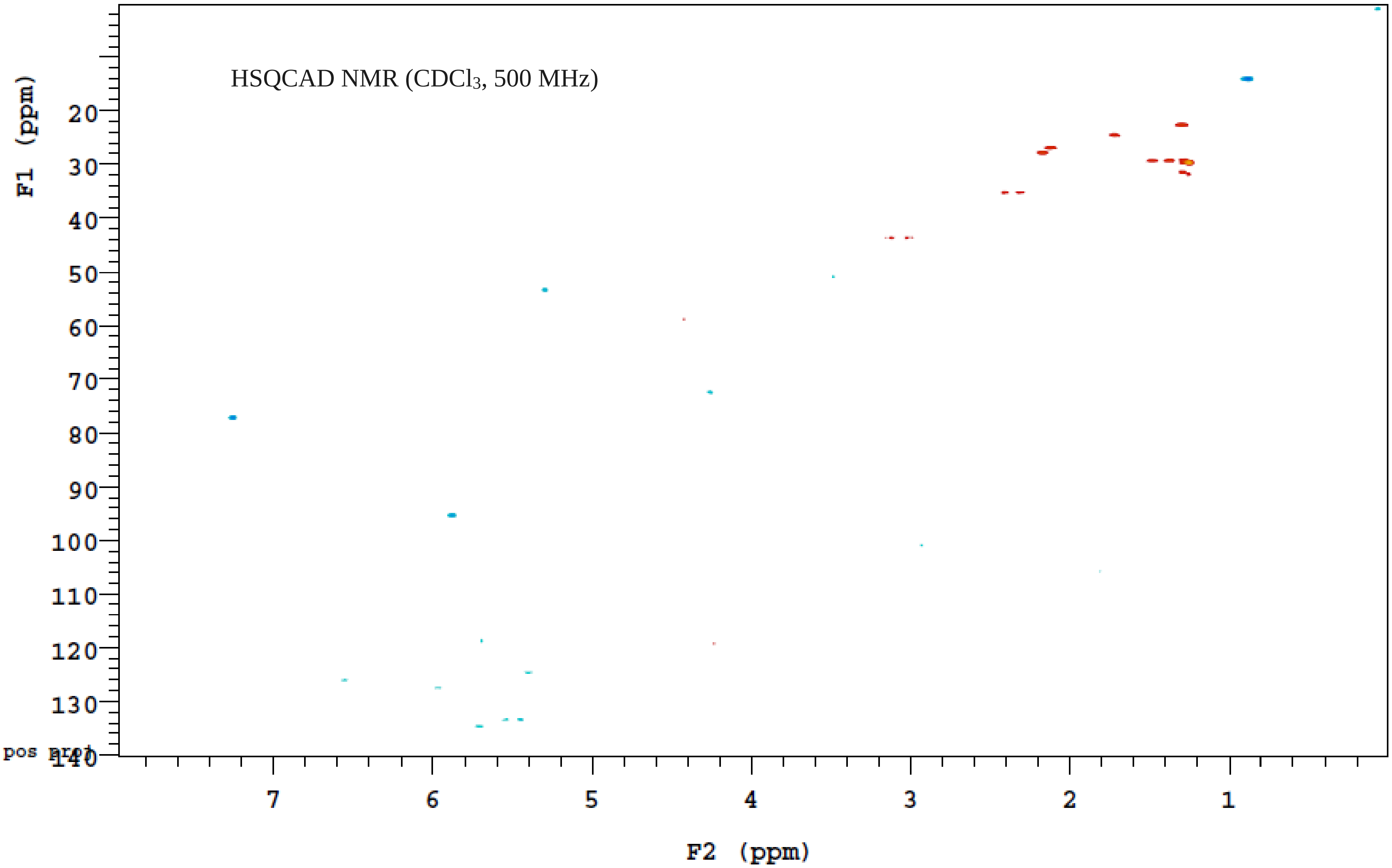

S9. HSQCAD NMR spectrum (500 MHz, $CDCl_3$) of retroflexanone (**1**).

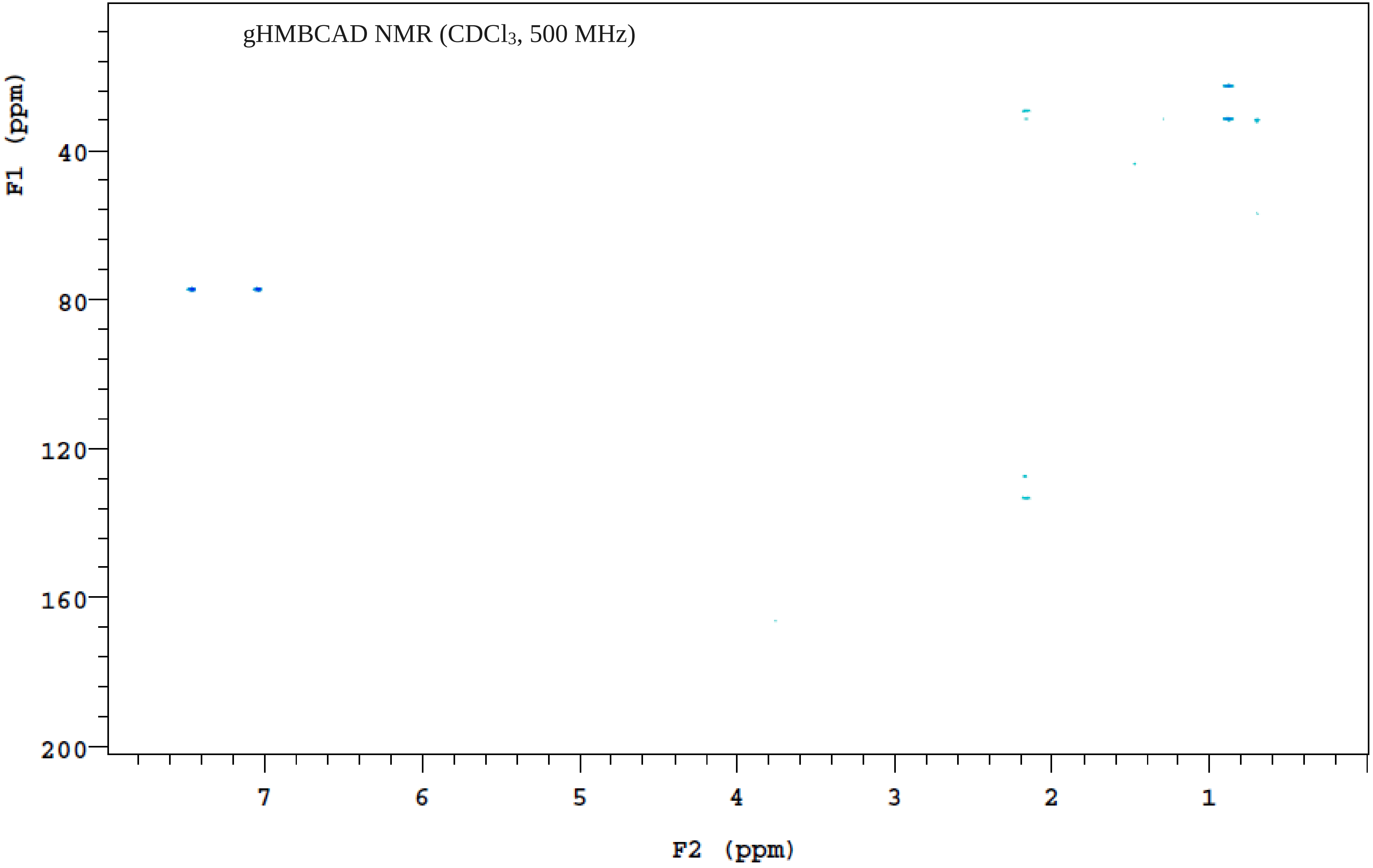

S10. gHMBCAD NMR spectrum (500 MHz, $CDCl_3$) of retroflexanone (**1**).

S11. Table of NMR data of retroflexanone (**1**).

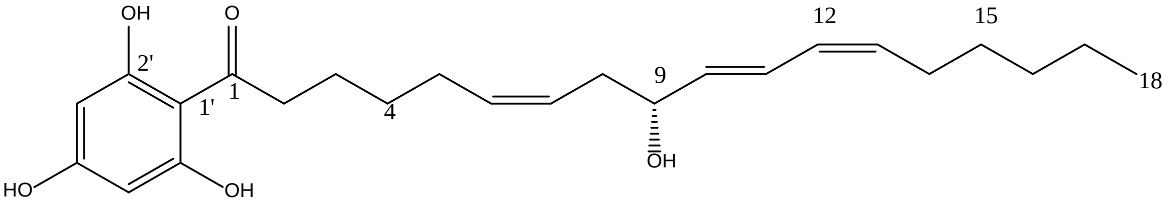

Position	Carbon, type[a]	Proton, mult. (*J* in Hz)	gCOSY	gHMBCAD
1	ND			
2a	43.6, CH_2	3.14, m	2b, 3[w]	
2b		3.00, m	2a	
3	24.4, CH_2	1.72, m	2a, 2b, 4[w]	
4	29.3, CH_2	1.48, m	3, 5	2
5	26.8, CH_2	2.12, m		
6	133.5, CH	5.54, m	7	
7	124.7, CH	5.39, m	6, 8b	
8a	35.2, CH_2	2.41, m		
8b		2.31, m	7, 9	
9	72.5, CH	4.26, dt (6.0, 6.5)	8a, 8b, 10	
10	134.6, CH	5.70, dd (6.0, 15.0)	9, 11	
11	126.1, CH	6.52, dd (11.5, 15.0)	10, 12	
12	127.5, CH	5.97, dd (11.0, 11.5)	11, 13, 14[w]	
13	133.4, CH	5.45, dt (7.0, 11.0)	12, 14	
14	27.4, CH_2	2.17, m	12[w], 13, 15	12, 13, 15, 16
15	29.3, CH_2	1.37, m	14, 16	
16	31.4, CH_2	1.29, m	15	
17	22.6, CH_2	1.29, m	18	16
18	14.1, CH_3	0.88, t (7.0)	17	16, 17
1’	ND			
2’	ND			
3’	95.4, CH	5.88, s		
4’	ND			
5’	95.4, CH	5.88, s		
6’	ND			
9-OH		ND		
2’-OH		ND		
4’-OH		ND		
6’-OH		ND		

Referenced to $CDCl_3$, 500 MHz

[a] Carbon assignments based on HSQCAD and gHMBCAD NMR experiments

[w] indicates weak or long rang correlation

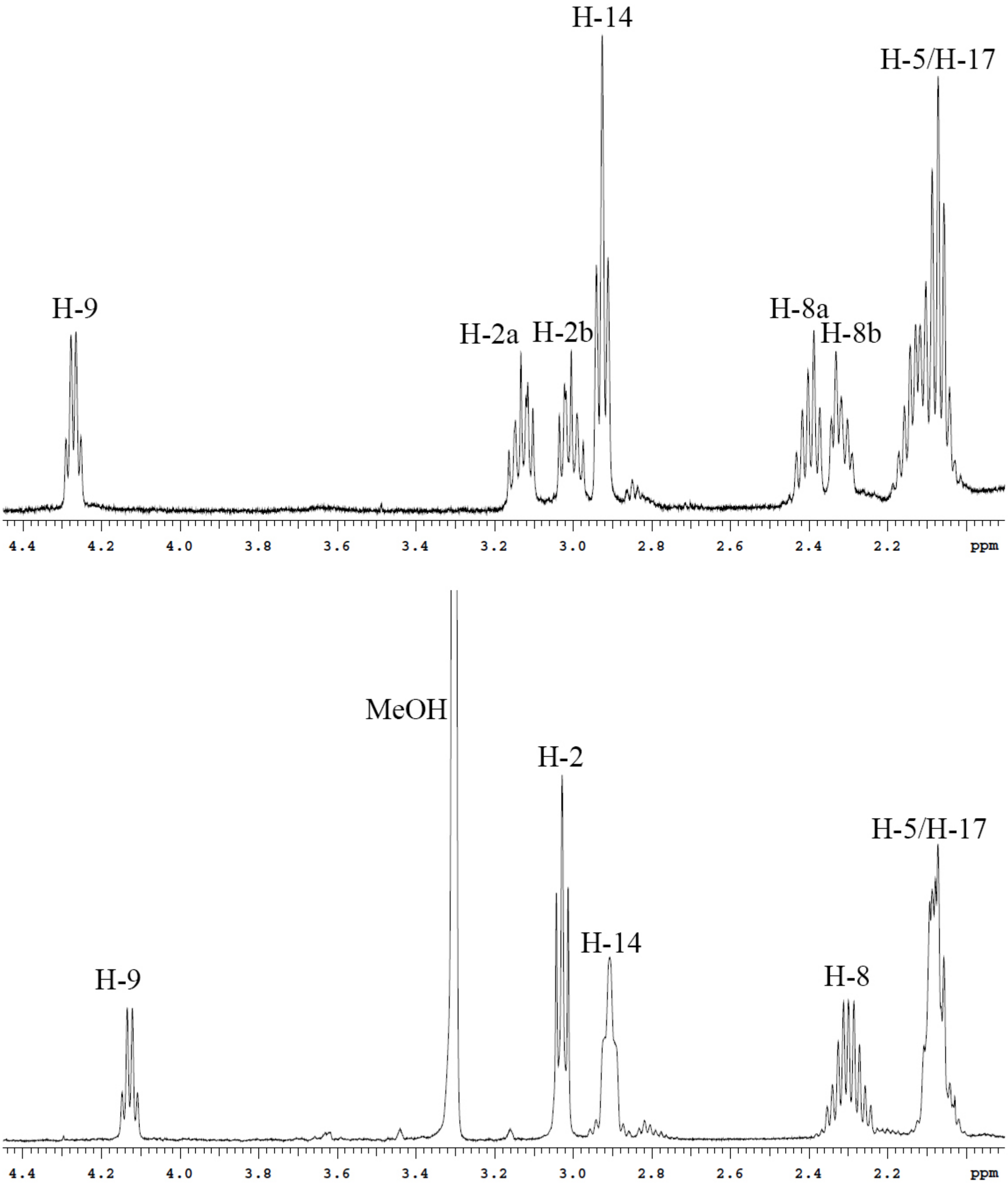

S12. Comparison of the upfield region of the ^{1}H NMR spectra of **2** in $CDCl_3$ (**top**) and CD_3OD (**bottom**).
